# Integrated miR-omics and proteomics reveal the regulatory role of miR in protein networks associated with COVID-19 disease progression

**DOI:** 10.3389/fimmu.2026.1796638

**Published:** 2026-09-14

**Authors:** Kelly E. Mercer, Vikrant Vijay, Li-Rong Yu, Tao Han, Elysia A. Masters, Jinchun Sun, Mallikarjun Bidarimath, Megan Ramey, Mona Agrawal, Armando S. Flores-Torres, Amanda M. Green, Keith Burkhart, Chistian Herzog, Giuseppina Dusio, John M. Arthur, Jessica Oliphant, Heather S. Smallwood, Richard D. Beger

**Affiliations:** 1 Division of Systems Biology, National Center for Toxicological Research, United States Food and Drug Administration, Jefferson, AR, United States; 2 Department of Pediatrics, University of Tennessee Health Science Center, Memphis, TN, United States; 3 Department of Infectious Diseases, St Jude Children’s Research Hospital, Memphis, TN, United States; 4 Division of Applied Regulatory Sciences, Office of Clinical Pharmacology, Office of Translational Sciences, Center for Drug Evaluation and Research, United States Food and Drug Administration, Silver Spring, MD, United States; 5 Department of Nephrology, University of Arkansas for Medical Sciences, Little Rock, AR, United States; 6 Department of Pathology and Genomic Medicine, Houston Methodist Research Institute, Houston, TX, United States

**Keywords:** COVID-19, miRNA profiling, multiomics, pathway, proteomics

## Abstract

**Introduction:**

While microRNA (miR) expression profiling has identified potential biomarkers in patients with COVID-19, the regulatory mechanisms by which miRs modulate disease severity remain poorly characterized. We performed integrated miR–proteome analysis to elucidate mechanistic relationships between miR regulation and COVID-19 severity.

**Method:**

Deidentified plasma samples from 93 participants with acute COVID-19 were categorized by severity using a 12-point symptom scoring system: mild (0–1), moderate (2–4), and severe (5–12). miR and proteomic profiles were analyzed using univariate statistics, pathway analysis, miR-target prediction, and correlation analysis. Differentially expressed miRNAs (DEMs) and differentially expressed proteins (DEPs) between the three severity groups from the original cohort were evaluated in a validation cohort of 94 participants.

**Results:**

We identified 365 unique miRs and 801 unique proteins that were significantly associated with COVID-19 severity in any of the three comparisons. Ingenuity pathway analysis revealed neutrophil degranulation, cytokine storm, interleukin-10 (IL-10) signaling, and wound healing signaling as top dysregulated pathways in severe versus mild cases, with IL-6 involved in 9 of the 10 most significant pathways. Correlation analysis between miRs and proteins from 93 participants identified 6,559 miR–protein pairs with |*r*| > 0.5, of which 83.6% were negative correlations, suggesting widespread miR-mediated downregulation of protein expression. A significant correlation (*r* = 0.43, *p* < 0.0001) was found between 122 predicted miR–protein pairs found in the discovery cohort and the same miR–protein pairs in the validation cohort.

**Discussion:**

To our knowledge, this represents the first integrated analysis of circulating miRs and proteins from the same COVID-19 participants and separately in a validation cohort. The high frequency of negative miR–protein correlations combined with target prediction analysis suggests that miRs play regulatory roles in COVID-19 severity-associated pathways. These findings provide mechanistic insights into miR regulation of host immune responses and identify potential biomarkers that could inform therapy of COVID-19.

## 1 Introduction

SARS-CoV-2 infections and its subsequent disease, COVID-19, have had a devastating global impact, causing over 777 million infections and 7 million deaths worldwide (COVID-19 cases | WHO COVID-19 dashboard). While the acute phase of the pandemic has subsided, the virus continues to pose a significant health challenge, highlighting the need to understand the factors contributing to varying disease severity. Age, sex, and underlying health conditions such as diabetes, cancer, obesity, high blood pressure, asthma, and smoking have been identified as risk factors for severe COVID-19 outcomes (1–4). However, these factors alone do not fully explain the heterogeneity of patient responses. Gaining mechanistic insights at the molecular level is crucial for understanding how these risk factors translate into severe disease and for developing targeted therapies to improve patient outcomes.

A hallmark of severe COVID-19 is the “cytokine storm”, a dysregulated immune response characterized by excessive production of pro-inflammatory cytokines (5–10). Cytokines play critical roles in cell signaling, immune regulation, and tissue damage. To elucidate the complex interplay of factors involved in patient responses to SARS-CoV-2 infection, numerous proteomics studies have sought to identify protein biomarkers associated with COVID-19 (11, 12) and disease severity (13–18). MicroRNAs, small noncoding RNA molecules that regulate gene expression, have also emerged as key players in immune responses (19–22). Profiling microRNA (miR) expression patterns in patients with COVID-19 has revealed potential miR biomarkers associated with disease outcomes (8, 23–30) and reported in meta-reviews (31, 32). While multiomics studies have begun to unravel the complexities of COVID-19 responses (33, 34), a comprehensive integration of proteomics and miR-omic profiling from the same individuals remains limited.

To address this gap, we performed miR profiling and proteomics analysis of plasma samples from a discovery cohort of 93 SARS-CoV-2-infected participants, collected between the summer of 2020 and the summer of 2022, and validated our miR, protein, and miR–protein relationship findings in an independent cohort of 94 patients. Patient responses were classified based on a 12-point scoring system, categorizing them as mild (0–1), moderate (2–4), or severe (5+) (35). The goal of this study is to discover miR and protein biomarkers and elucidate molecular mechanisms associated with severity of response to COVID-19, with the ultimate aim of facilitating more effective diagnostic, therapeutic, and risk assessment approaches as SARS-CoV-2 becomes integrated into the landscape of seasonal infectious diseases.

The primary novelty of this work lies not simply in the co-profiling of miRNAs and proteins from the same individuals, but in the analytical framework used to integrate these data. By combining ingenuity pathway analysis (IPA)-based miR-target prediction with large-scale Pearson correlation analysis between differentially expressed miRNAs (DEMs) and proteins, we move beyond biomarker identification to propose specific regulatory relationships that may play a role in COVID-19 severity. Critically, this approach uses the correlation data to empirically filter and prioritize predicted miR–protein interactions—retaining only those pairs supported by both computational prediction and observed co-expression patterns in plasma. This dual-evidence framework reduces reliance on any single method, each of which carries known limitations: target prediction models are limited by the accuracy of the predictions, while correlation alone cannot establish the directionality of regulation. Together, they converge on a set of miR–protein pairs with mechanistic plausibility and statistical support, providing a ranked list of candidates for future therapeutic investigation. To our knowledge, this represents the first study to apply this integrated strategy to simultaneously profile miRNA and protein expression at this scale in two independent patient cohorts of SARS-CoV-2-infected individuals.

## 2 Methods

### 2.1 Study cohorts, recruitment, and response group assignment

In the initial cohort, participant plasma samples were collected as part of the Clinical, Immunological and Virological Characterization of COVID-19 (CIVIC-19) study (35). The clinical studies were approved by the Institutional Review Boards of the University of Tennessee Health Science Center (UTHSC) and Univerisity of Arkansas for Medical Sciences (UAMS). Plasma samples were collected from 93 SARS-CoV-2-positive participants (47 women and 46 men), confirmed via nasal swab polymerase chain reaction (PCR) testing, with ages ranging from 16 to 78 years. Samples were obtained during the acute phase of infection, on the day participants enrolled in the study. Participants were recruited from inpatient hospitals and outpatient testing centers located in Memphis, Tennessee between August 2020 and July 2022.

Written informed consents were obtained from all participants. Demographics, medical history, any COVID-19 symptoms, effects, and treatments were collected from electronic medical records and participant questionnaires.

The scoring followed Modified World Health Organization case definition based on COVID-19 severity scoring criteria, as described earlier (35): (i) two points for Hospital Admission within 60 days of enrollment; (ii) three points for intensive care unit (ICU) admission within 60 days of enrollment; (iii) one point for mild symptoms including cough, fever, diarrhea, vomiting, headache, loss of taste or smell, sore throat, myalgias, fatigue, lymphadenopathy, and malaise; (iv) two points for moderate symptoms including shortness of breath (dyspnea), wheezing, SpO_2_ (oxygen saturation) <92% on room air, respiratory rate (RR) > 30, and new non-invasive oxygen requirement; (v) two points for severe symptoms: invasive or positive-pressure oxygen requirement, acute kidney injury [creatinine >1.5× upper limit of normal for age or estimated glomerular filtration rate (eGFR) < 60], elevated aspartate/alanine transaminase (AST/ALT) ratio >2× normal, new elevation international normalized ratio (INR) >1.3, and altered mental status; and (vi) three points for critical symptoms including acute respiratory distress syndrome (ARDS), shock requiring vasopressors, renal failure with dialysis, extracorporeal membrane oxygenation (ECMO) requirement, requirement of organ transplant, pulmonary embolism, deep venous thrombosis, and stroke.

Patient selection of the original cohort was conducted to have approximately 30 participants in each severity response group with similar numbers of men and women in each response group. Plasma samples were aliquoted and deactivated by incubating in a 56 °C water bath for 30 min for proteomics and for 45 min for miR-omics analyses, then stored at −80 °C at UTHSC prior to shipping to US FDA’s National Center for Toxicological Research (NCTR). The plasma samples from UTHSC were used to generate original data, and a second set from UAMS participants was used to generate validation data. The same methods were applied to both original and validation samples.

In the second, validation cohort, 94 plasma samples were collected at UAMS in an IRB-approved study titled “ACE 2 Enzyme to Predict Outcomes in Infection with SARS-CoV-2” and used as a validation cohort. The samples were collected before treatment for COVID-19, and SARS-CoV-2 infection was confirmed by PCR. All the plasma samples were collected using green (Li-heparin) top tubes. UAMS IRB approval was obtained for each for collection and shipment of samples to NCTR. Patient demographics for age, sex, and race was provided. A total of 65 patients were categorized into three symptom response categories using the WHO scoring criteria described previously and 29 patients using the criteria listed below.

1. Mild: Outpatients: Asymptomatic. None of the symptoms described below and any of the following symptoms: Fever (>100.4°F), cough, sore throat, myalgia, or fatigue that do not require hospitalization.
2. Moderate: Hospitalization without intensive care: Any of the above symptoms plus shortness of breath or oxygen saturation ≤88% at rest or tachypnea (≥30 breaths per min), or requirement for supplemental oxygen.
3. Severe: Hospitalization with intensive care: Respiratory failure requiring mechanical ventilation or septic shock, or other organ dysfunction or failure that requires intensive care.

Validation samples from UAMS were prepared for shipment to NCTR using the same methods that were used in the original samples from UTHSC.

### 2.2 MiR methods

Plasma samples (33 μL) were shipped and used to prepare miR libraries, and miRseq was performed by Firalis S. A. (Huningue, France) using HTG EdgeSeq technology (HTG Molecular Diagnostics, Tucson, AZ, USA) that uses EdgeSeq miR whole transcriptome assay to enrich the miRs without RNA extraction, followed by Illumina HiSeq 4000 sequencing to profile 2083 miRs (36). The sequencing steps involved in EdgeSeq miR whole transcriptome assay include HTG EdgeSeq analysis, molecular barcoding and adapter addition, PCR clean-up, determination of library concentration, sample pooling and sequencing, and sequence analysis. In the end, barcoded samples were sequenced using HiSeq 4000 (Illumina) with FASTQ-only output. Six pooled plasma samples were used for QC purpose of miRNAs in this study. Assay results for 2,083 miRs were reported as raw counts for 92 samples.

### 2.3 Protein methods

Deactivated plasma samples (80 μL) were shipped and analyzed for protein expression by Olink Proteomics (Boston, MA) using the Proximity Extension Assay technology (37). Analysis was conducted for the Olink^®^ Explore 3072 panel, which was a combination of eight separate Explore 384 panels, namely, inflammation, oncology, cardiometabolic, neurology, inflammation II, oncology II, cardiometabolic II, and neurology II. Quality control was performed using Olink in-house control samples and seven pooled plasma samples. Assay results for 2,921 proteins were reported in arbitrary, relative units as Normalized Protein eXpression (NPX) for 93 samples. More information regarding the Olink platform, NPX, assay validation data, and the full list of proteins in the panel is available at Olink’s website (www.olink.com).

### 2.4 Data analysis methods

#### 2.4.1 Patient demographic analysis method

Continuous variables of patient demographics were expressed as mean with standard deviation. Categorical variables of patient underlying health conditions and ICU sickness were expressed as frequencies and percentages of participants with known health/sickness conditions. Participants with unknown underlying health conditions and ICU sickness were not included in these categories. The differences between COVID-19 disease severity groups were evaluated using *t-*test for continuous variables and chi-square test (Fisher’s exact test if the count was <5) for categorical variables, where *p*-values <0.05 were considered statistically significant.

#### 2.4.2 miR and protein data analysis

Expression profiling of 2,083 miRs from 92 plasma samples were performed using the HTG EdgeSeq system, and raw counts of miRs were obtained. A total of 2,921 proteins from 93 plasma samples were analyzed using Olink Explore 3072 proteomics assays and NPX values were obtained. All data were imported into SAS system for Windows, version 9.4(TS1M7) (SAS Institute Inc., Cary, NC, USA) for data analysis using SAS/STAT 15.2 software. The raw counts of miRs were converted into count per million (CPM) values for each sample and subsequent 75th percentile normalization was performed to make the distribution of data equal in all samples. Both normalized miR and proteomic data were log2 transformed before statistical analysis. Correlation between samples within group and pooled samples was performed to evaluate robustness of data. General Linear Model procedure (proc glm) with estimate statement within SAS/STAT software was used to compare data to obtain log2 mean and statistical significance [*p*-values and false discovery rate (FDR)]. Fold changes between different COVID-19 response groups was calculated by taking anti-log2 of log2 mean. The coefficient of variation was calculated for each miR/protein using six to seven pooled sample data and set to 50% to limit the influence of highly variable analytes. miRs with a fold change (FC) >2, *p*-value <0.05, and coefficient of variation (CV) <50% were defined as DEMs. Proteins with FC >1.5, *p*-value <0.05, and CV <50% were defined as differentially expressed proteins (DEPs), as described in the literature (38, 39). Guo et al. (38) demonstrated that a combined fold change and *p*-value (FC > 2.0 + PV <0.05) approach performs well for identifying transcriptomic features for downstream pathway analysis. FDR-adjusted *p*-values were calculated for all analytes and are reported in **Supplementary Tables 2** and **3**. However, differential expression was defined using the pre-specified FC+PV+CV framework to prioritize analytes exhibiting both consistent statistical evidence and biologically meaningful effect sizes. This approach was selected as *a priori* to facilitate pathway discovery and candidate prioritization and was subsequently evaluated using an independent validation cohort. Comparison of analytes identified using the FC+PV+CV framework with those meeting FDR-based significance criteria demonstrated substantial overlap. We found that 98.5% proteins that were significant using PV+FC+CV had an FDR < 0.1 in the severe vs. mild discovery cohort and 96.0% of miRs that were significant using PV+FC+CV had an FDR < 0.1 in the severe vs. mild discovery cohort, thus supporting the robustness of the selected feature sets while preserving analytes with biologically meaningful changes for pathway analyses and validation studies. Pearson correlations between DEMs and DEPs were performed within base SAS software using correlation procedure (proc corr).

#### 2.4.3 Pathway analysis, miR–mRNA target predictions, and Pearson correlations

All protein data were imported into web-based IPA software (QIAGEN Inc., https://digitalinsights.qiagen.com/IPA). Core analysis of DEPs was performed to evaluate pathway enrichment using Fisher’s exact test. Canonical pathways with −log10 *p*-value >1.3 were considered significantly enriched. IPA also calculates *z*-scores to predict the activation/inhibition of pathways based on fold changes of altered proteins. *z*-score > 2 or *z*-score < −2 indicate predicted activation or inhibition of pathways, respectively.

Information about miR and their experimental or predictive biological targets was obtained from IPA using the “miR Target Filter” and various databases (TargetScan, miRecords, and miRTarBase) or literature (Ingenuity Expert Findings). Each protein target of a miR is assigned a confidence score by IPA as experimentally observed or high-, medium-, and low-predicted, and includes source material for the confidence prediction when applicable (40). IPA ranks predictive protein targets of miR using the cumulative weighted context score (CWCS) calculated by TargetScan’s published methodology (41).

Following miR–protein target prediction analysis, Pearson correlations were performed using base SAS correlation procedure (proc corr) between the unique miRs and proteins identified to have an association using IPA. A workflow outlining statistical analysis, pathway analysis, miR–protein target analysis, and correlations is illustrated in **Figure 1**.

**Figure 1:**
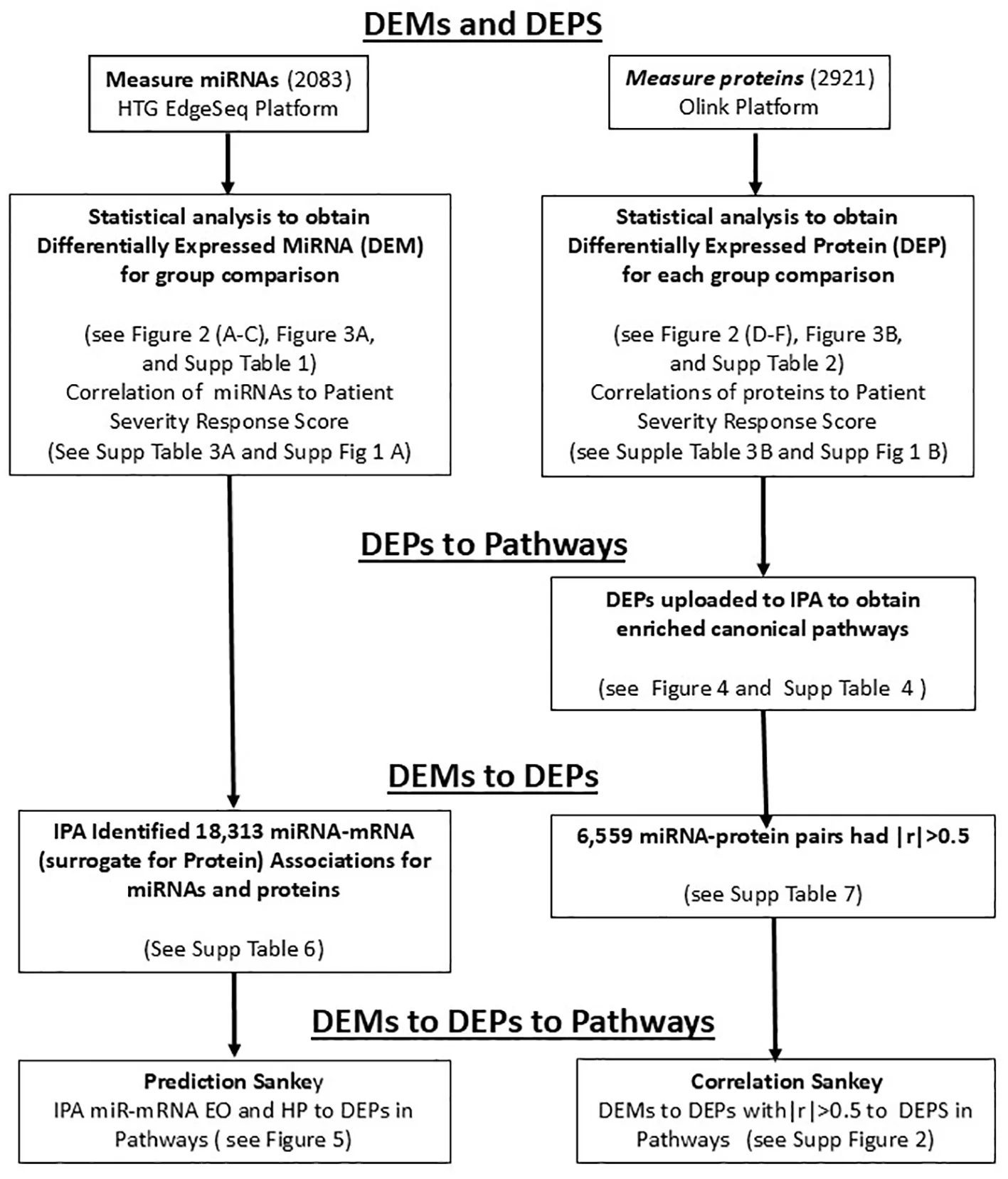
A flowchart of integrated miR–protein analysis.

### 2.5 Visualization methods

Volcano plots and partial least squares (PLS) plots were generated using SAS 9.4 software. Venn and chord diagrams were created using the “GOplot” (v1.0.2) package and heatmaps were created using “ComplexHeatmap” (v2.18.0) and “dendextend” (v1.17.1) packages in Rstudio 2023.03.0 Build 386 (R version 4.3.0). Sankey diagrams were created using the web resource “SankeyMATIC” (https://sankeymatic.com/). All other graphs were created in GraphPad Prism 10 (v10.4.1).

### 2.6 Reproducibility methods

The UAMS cohort of 94 individuals was used for reproducibility. The validation cohort was analyzed for patient demographics of age, sex, and race between the response groups. The DEMs and DEPs discovered in the UTHSC cohort were evaluated in the UAMS cohort using the same thresholds. The common DEPs in the UAMS validation cohort were evaluated by pathway enrichment in IPA. The UTHSC miR–protein pairs in the UTHSC discovery cohort were evaluated in the UAMS validation cohort using both IPA prediction of miR targets and correlation between miR and proteins in the cohort. Pearson correlations were conducted to validate miR–protein pairs identified in the UTHSC discovery set with the miR–protein pairs in the UAMS validation set.

## 3 Results

### 3.1 Patient demographic results

The initial UTHSC cohort included 93 individuals who tested positive for SARS-CoV-2 by real-time RT-PCR methods. These participants were classified into mild (*N* = 33, 45.5% female), moderate (*N* = 28, 57.1% female), and severe (protein: *N* = 32, 50.0% female) COVID-19 infection groups based on the modified WHO severity scoring criteria (**Table 1**). There were no significant differences in sex, underlying health conditions (i.e., diabetes, hypertension, cardiovascular disease, obesity, and chronic renal or lung disease), or vaccinated status across all three groups. However, race and age were significant factors in the comparisons between severe and mild response groups.

**Table 1:** Demographics of participants by COVID-19 severity.

Characteristic	Patient response category			Severity comparison		
	Mild (*N* = 33)	Moderate (*N* = 28)	Severe (*N* = 32)	^1^*p*-value	^2^*p*-value	^3^*p*-value
Severity score	0.79 ± 0.42	2.82 ± 0.98	8.50 ± 2.24	<0.0001	<0.0001	<0.0001
Age	39.3 ± 14.3	44.6 ± 14.4	52.2 ± 19.0	0.0032	0.086	0.155
Female	15 (45.5)	16 (57.1)	16 (50.0)	0.714	0.580	0.363
Black	3 (9.1)	10 (35.7)	22 (68.8)	<0.0001	0.010	0.014
White	7 (21.2)	5 (17.9)	9 (28.1)	0.518	0.348	0.743
Other race	0 (0.0)	2 (7.1)	1 (3.1)	0.492	0.594	0.207
Race unknown	23 (69.7)	11 (39.3)	0 (0.0)	<0.0001	<0.0001	0.017
Underlying health conditions						
Diabetes	4/13 (30.8)	5/15 (33.3)	11/27 (40.7)	0.730	0.636	1.000
Hypertension	6/13 (46.2)	10/16 (62.5)	20/27 (74.1)	0.083	0.424	0.379
Cardiovascular diseases	3/13 (23.1)	8/16 (50.0)	10/27 (37.0)	0.484	0.405	0.249
Chronic renal disease	1/13 (7.7)	0/14 (0)	7/27 (25.9)	0.236	0.075	0.482
Severe obesity	3/12 (25.0)	8/16 (50.0)	11/27 (40.7)	0.477	0.555	0.253
Chronic lung disease	0/13 (0)	3/14 (21.4)	7/27 (25.9)	0.074	1.000	0.222
Vaccinated status	7/32 (21.9)	6/27 (22.2)	7/30 (23.3)	0.891	0.921	0.974

For Scoring and Age, data (continuous) are presented as mean ± SEM; for the rest of the characteristics, data are presented as counts (%). Differences between severity groups were evaluated using *t-*test for continuous variables and chi-square test (Fisher’s exact test if the count is <5) for categorical variables; significance *p* < 0.05. ^1^Severe vs. Mild, ^2^Severe vs. Moderate, ^3^Moderate vs. Mild.

### 3.2 Plasma miR and protein expression signatures discriminate between COVID-19 severity groups

Differential expression analysis was performed to identify significant DEMs and DEPs within the three severity group comparisons, moderate vs. mild, severe vs. moderate, and severe vs. mild, in the original discovery data. Volcano plots depicted in **Figure 2** highlight these findings. The *x*-axis represents the log2 fold change and the *y*-axis represents the log10 *p*-value. With respect to miR, 84 DEMs were identified in the moderate vs. mild comparison, of which 88% had decreased expression; 195 DEMs were identified in the severe vs. moderate comparison, of which the majority (84%) had increased expression; and 273 DEMs were observed in the severe vs. mild comparison, of which 147 had decreased expression (54%) and 126 had increased expressions (**Figures 2A–C**). Overall, 365 unique DEMs (in any of three comparisons) were identified in the discovery dataset. With respect to proteins, we identified 69 DEPs in the moderate vs. mild comparison, with 74% of these having increased expression. Similarly, 503 and 742 DEPs were identified in the severe vs. moderate and severe vs. mild comparison groups, respectively (**Figures 2D–F**). In both comparison groups, the majority (~90%) had increased expression. Overall, 801 unique DEPs (in any of three comparisons) were identified in the discovery dataset. **Supplementary Tables 2** and **3** provide more detailed information on univariate and group comparison statistics (*p*-value, fold change, FDR, and CV) for miRs and proteins, respectively.

**Figure 2:**
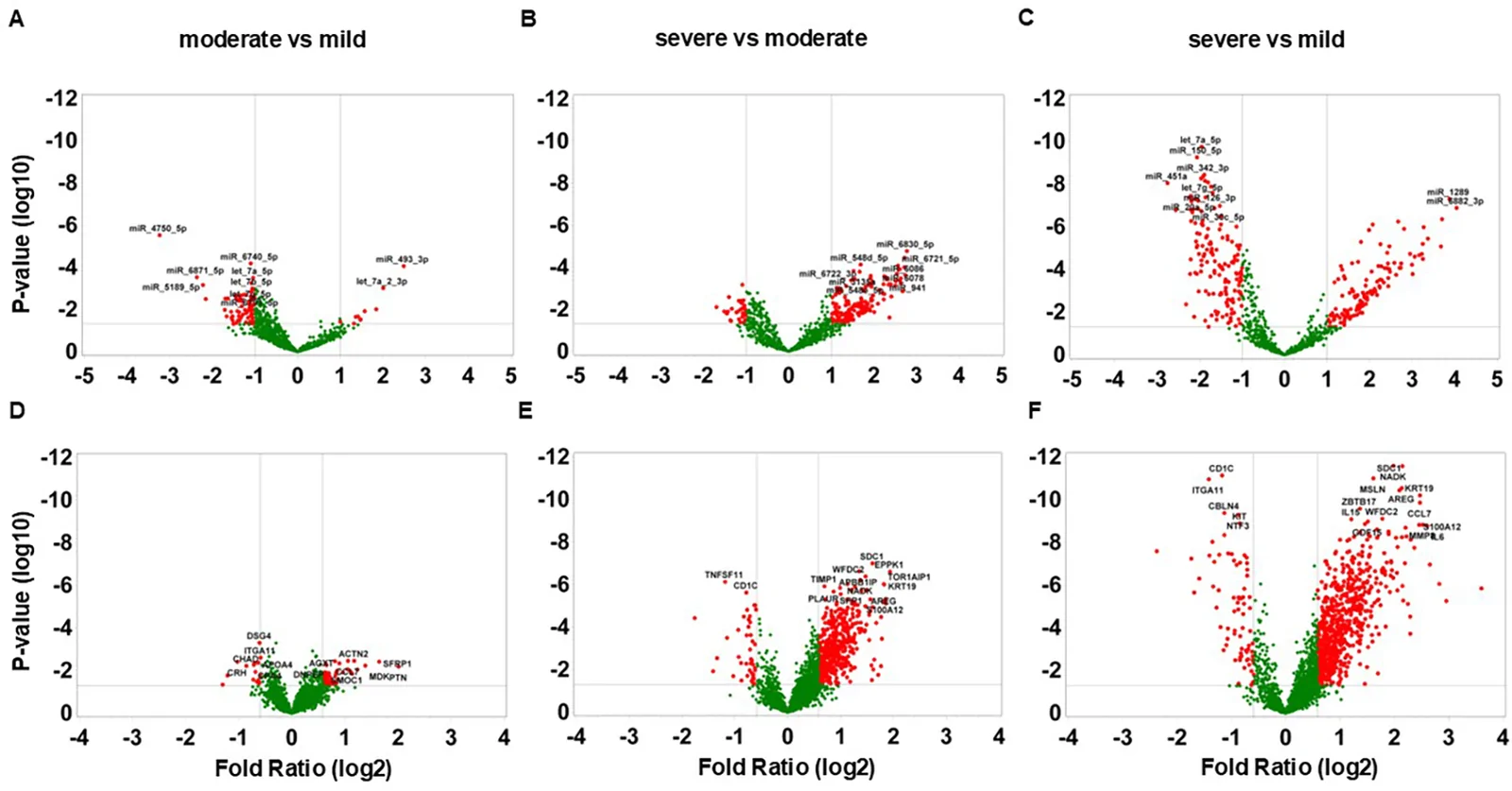
Volcano plots show the total miR and protein expression profiles for a given pair of comparisons. For miRs **(A–C)** and proteins **(D–F)**, DEMs and DEPs are separated by gray lines located at the cutoff values established using the following parameters: DEMs, FC > 2.0, and *p*-value < 0.05; DEPs, FC > 1.5 and *p*-value < 0.05 are shown as red dots, and non-significant values are shown as green dots.

The overlap of significant DEMs and DEPs among the three comparison groups was analyzed and presented as Venn diagrams in **Figures 3A, B**. As expected, the severe vs. mild comparison group had the most unique DEMs (18 increased and 99 decreased) and DEPs (227 increased and 33 decreased), followed by the severe vs. moderate and moderate vs. mild comparisons. Moreover, 15 miRs and 27 proteins were identified at the intersection as potential discriminators of COVID-19 severity. Heatmaps showing the fold change differences of these discriminating miRNAs and proteins across all three comparison groups are presented in **Figures 3A, B**. These cursory analyses were consistent with literature consensus that pro-inflammatory responses/pathways drive COVID-19 severity. For example, several downregulated miRs listed in **Figure 3A**, including miR-17-5p, miR-20a-5p, miR-106a-5p, and miR-451a, have been previously identified to be markers for COVID-19 severity and progression (42). With respect to proteins, interleukin-6 (IL-6) and hepatocyte growth factor (HGF) had been reported to predict severity and mortality in patients with COVID-19 (43–47). Additional pro-inflammatory markers previously reported to characterize disease severity (35) were also significantly increased in the severe vs. moderate and/or severe vs. mild comparison groups. These included interleukin-8 (IL-8, also known as CXCL8), interferon-γ-induced protein 10 kDa (IP-10, also known as CXCL10), interleukin-1 receptor antagonist (IL-1RN), macrophage inflammatory protein-1β (MIP-1β, also known as CCL4), and chitinase-3-like protein 1 (CHI3L1). In contrast, other reported markers such as tumor necrosis factor-α (TNFα, also known as TNF), interleukin-1β (IL-1β), and interleukin-10 (IL-10) did not change between severity group comparisons (**Supplementary Table 3**).

**Figure 3:**
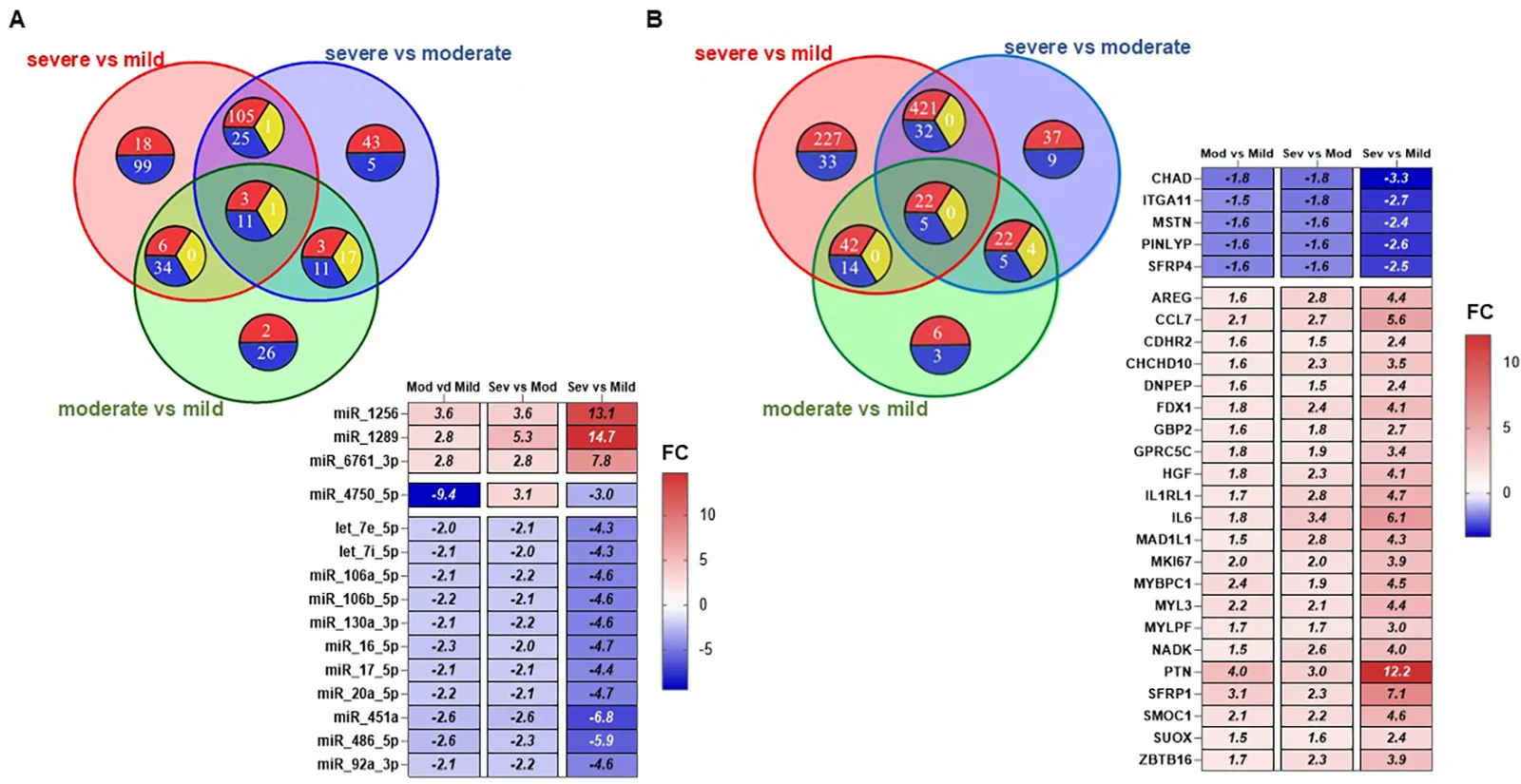
Venn diagrams show the overlap of significant DEMs and DEPs between severity group comparisons. Venn diagrams for **(A)** DEMs and **(B)** DEPs and heatmaps displaying fold change expression of common miRs and proteins at the intersection of moderate vs. mild, severe vs. moderate, and severe vs. mild comparison groups. For Venn diagrams, red shaded circle, severe vs. mild; blue shaded circle, moderate vs. mild; and green shaded circle, moderate vs. mild; smaller circles show consistent increased (red) and decreased (blue) protein expression with the comparison groups. The yellow color in the small circles of the intersecting response regions lists the number of changes that are in different directions in the two regions.

To gain further insights, Pearson’s correlations were evaluated between the expression levels of each downregulated DEM and each upregulated DEP identified in the severe vs. mild comparison and their respective severity scores across all samples. The analysis revealed 132 negatively correlated miRs and 644 positively correlated proteins (FDR < 0.05), with −log10 *p*-values plotted and categorized by correlation strength: strong (*r* < −0.5), moderate (−0.3 > *r* > -0.5), and weak (*r* > −0.3) (**Supplementary Figure 1A**). The miR most negatively correlated with severity (**Supplementary Table 4A**) was miR-150-5p (*r* = −0.62, *p* = 4.5E−11), previously identified as a biomarker for severe pulmonary disease (48). Other miRs with established negative associations with COVID-19 severity, including miR-342-3p (*r* = −0.57, *p* = 7.8E−8) and miR-191-5p (*r* = −0.49, *p* = 5.6E−7), show similar correlations in our cohort (49, 50). Among the top 10 most significantly correlated proteins (**Supplementary Table 4B**), only Amphiregulin (AREG, *r* = 0.71, *p* = 1.1E−15) and mitotic arrest deficient 1 like 1 (MAD1L1, *r* = 0.73, *p* = 1.1E−16) were previously identified as significant discriminators in **Figure 3B**. Others among the top 10 were syndecan-1 (SDC1, *r* = 0.73, *p* = 1.0E−16) and keratin 19 (KRT19, *r* = 0.71, *p* = 1.7E−15), which mark endothelial and epithelial cell injury, respectively, and predict COVID-19 severity and mortality (51, 52). Additionally, pulmonary-specific markers for injury and tissue remodeling, i.e., mesothelin (MSLN, *r* = −0.70, *p* = 4.3E−15) and epiplakin 1 (EPPK1, *r* = −0.68, *p* = 2.8E−14) showed strong correlations (53, 54). Complete lists of DEMs and DEPs with significant correlations (FDR < 0.5) are provided in **Supplementary Tables 4A, B**.

### 3.3 Canonical pathways enrichment

To identify pathways involved in COVID-19 severity progression, IPA was used to cluster significant DEPs from each severity group comparison into biological pathways for the original dataset. A total of 6, 83, and 118 enriched canonical pathways were identified by applying a threshold of −log10 *p*-value > 2 and absolute (*z*-score) >2 in the moderate vs. mild, severe vs. moderate, and severe vs. mild comparisons, respectively (**Supplementary Table 5**). There are 5 pathways that are common to all three group comparisons and 68 pathways that are common to severe vs. moderate and severe vs. mild. **Figure 4** displays −log10 *p*-values and *z*-scores of the 10 most significant enriched pathways identified in the severe vs. mild group with corresponding values in other comparisons. The top *x*-axis presents the −log10(*p*-value) and the bottom *x*-axis displays the *z*-score showing pathway activation. Significant overlap was observed in pathway enrichment in the severe vs. mild and severe vs. moderate comparisons with neutrophil degranulation, the most significantly activated (*z*-score > 6). This finding is aligned with other COVID-19 studies that have demonstrated a role for activated neutrophils in promoting disease severity (55, 56). Within the top 10, pathogen-induced cytokine storm signaling was the only pathway enriched in all three comparison groups, with increasing significance from moderate vs. mild (−log10 *p*-value = 5.51, *z*-score = 2.0), severe vs. mild (−log10 *p*-value = 15.2, *z*-score = 4.71), and severe vs. moderate comparison groups (−log10 *p*-value = 14.6, *z*-score = 3.45).

**Figure 4:**
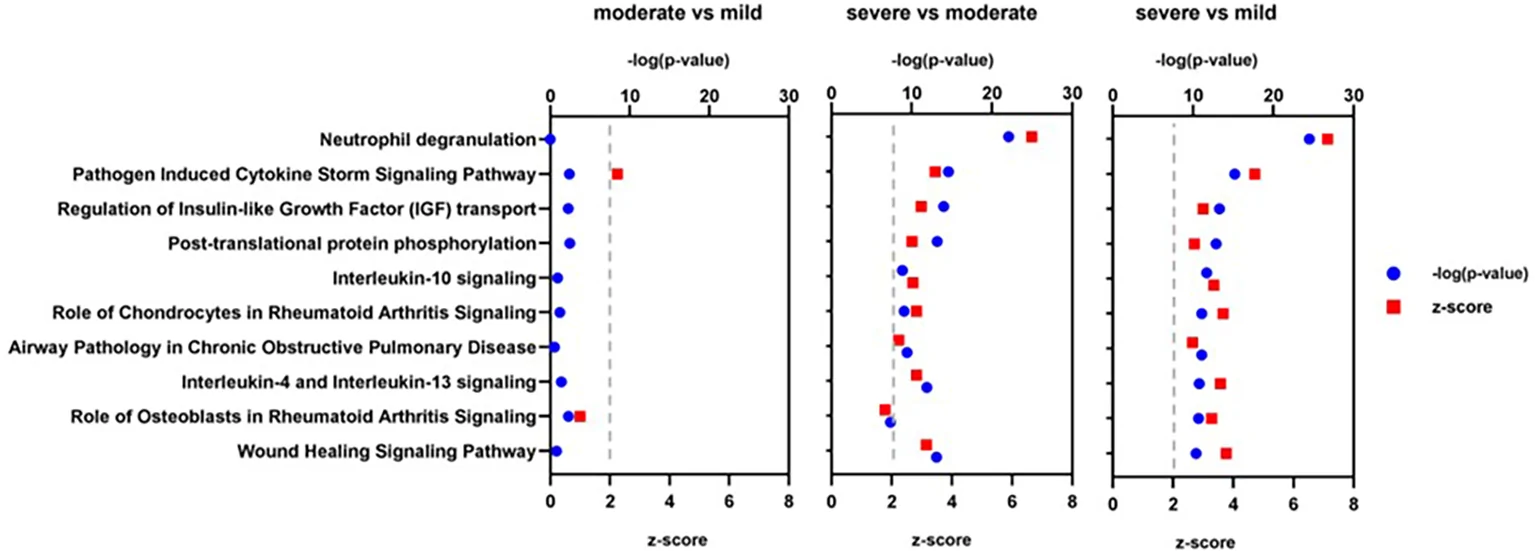
Canonical pathway enrichment analyses of DEPs associated with COVID-19 severity. Dot plots showing *p*-values and *z*-scores across the severity group comparisons of severe vs. mild, severe vs. moderate, and moderate vs. mild of the top 10 IPA pathways.

### 3.4 IPA miR–protein target analysis

To characterize the miR–protein interactions with predictive capacity for COVID-19 severity and broaden mechanistic understanding, we performed an integrated analysis of miRNA and protein datasets. Using IPA’s miR Target filter tool, we identified 18,313 miR–protein target relationships derived from the miRs and proteins listed in **Supplementary Tables 2** and **3**. Each pair met confidence thresholds classified as either experimentally observed (EO) or highly predicted (HP), as presented in **Supplementary Table 6**. From this dataset, 9,901 miR–protein pairs contained at least one differentially expressed element: either a DEM, DEP, or both from the original dataset. Specifically, these pairs included 169 unique DEMs, 702 unique DEPs, or combinations involving 163 unique DEMs and 537 unique DEPs. These 9,901 pairs were then filtered based on their predicted relationship with proteins associated with significantly enriched canonical pathways identified in the severe vs. mild comparison. The resulting predicted network of 122 miR–protein connections between 60 miRs, 58 proteins, and 29 pathways is displayed in **Figure 5** as a Sankey diagram. To facilitate interpretation, the 29 enriched pathways were organized into four higher-order biological modules: Cytokine & Innate Immune Signaling pathways; Chronic Inflammation, Autoimmunity & Neuroinflammation pathways; Fibrosis, Organ Pathology & Tissue Remodeling pathways; and Core Cell Signaling & Cancer Biology pathways. Notably, IL-6 protein was represented in 23 of the 29 enriched pathways and was present within all four pathway modules, reflecting its widespread involvement across inflammatory, remodeling, and cellular signaling pathways.

**Figure 5:**
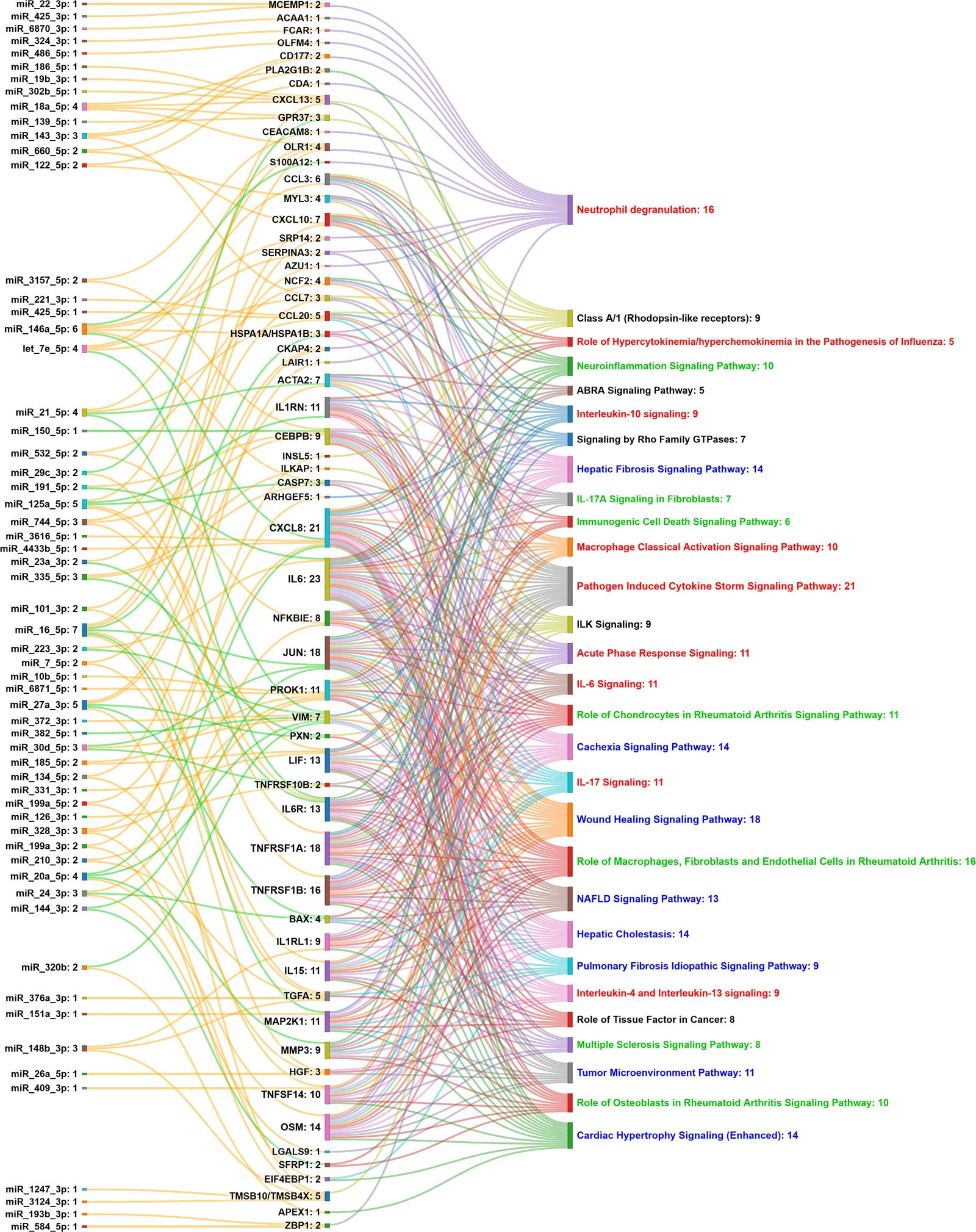
A Sankey diagram highlighting significantly enriched [−log10(*p*-value) > 4 and a *z*-score > 3] canonical pathways and associated significantly different (*p* < 0.05, FC > 2 in severe vs. mild comparison) proteins. Significantly different (*p* < 0.05, FC > 2 in severe vs. mild comparison) miR regulators (EO and HP; obtained from IPA) of these proteins are also shown. The experimentally observed (28) (shown in green lines) and high-predicted (94) (shown in orange lines) relationships between miRs and their protein targets. The pathways are color coded into four groups: red font for Cytokine & Innate Immune Signaling pathways; blue font for Chronic Inflammation, Autoimmunity & Neuroinflammation Pathways, green font for Fibrosis, Organ Pathology & Tissue Remodeling pathways; and black font for Core Cell Signaling & Cancer Biology pathways. There are 60 miRs, 58 proteins, and 29 pathways represented in this diagram. The references for the experimental observed miR–mRNA (protein) connections from IPA are provided (80, 92, 98–112). Fifty four of 60 miRNAs (8, 21, 23, 28–30, 32, 49, 50, 57, 58, 62, 64, 70, 79, 89, 113–152) and 55 of 58 proteins (11, 14, 43, 44, 47, 56, 66, 67, 69, 79, 137, 153–195) have been reported to be involved in COVID-19, and the references are provided as PMIDs in **Supplementary Table 7**.

Additional Pearson correlation analyses were conducted using original DEMs and DEPs from **Supplementary Tables 2** and **3**, yielding 363,300 miR–protein correlation measurements. From this dataset, 6,559 correlations between 141 miRs and 463 DEPs achieved absolute correlation values (*r*) above 0.5 while maintaining statistical significance (FDR < 0.05), with complete results provided in **Supplementary Table 7**. Most of these significant correlations (~84%) were inverse relationships. A Sankey diagram presented in **Supplementary Figure 2** depicts predicted network interactions for 967 negatively correlated miR–protein combinations associated with significantly enriched canonical pathways, regardless of IPA confidence level assignments. A comparative evaluation of miR–protein combinations identified through IPA prediction versus correlation analysis for three significant pathways, Neutrophil degranulation, Pathogen-induced cytokine storm signaling pathway, and Wound healing signaling pathway, is documented in **Supplementary Table 8**. Interestingly, when applying correlation coefficient information to the IPA prediction Sankey, only 13% of the miR–protein connections in the original data have strong negative correlations (*r* < −0.5). Nine miRNAs (miR-21-5p, miR-23a-3p, miR-199a-5p, miR-26a-5p, miR-16-5p, miR-24-3p, miR-30d-5p, miR-29c-3p, and miR-144-3p) and nine proteins (ACTA2, CCL7, HGF, IL-15, JUN, LGALS9, TNFRSF10B, TNFRSF1A, and SFRP1) were identified in both the IPA Sankey diagram (**Figure 5**) and the Correlation Sankey diagram (**Supplementary Figure 2**), and these miRs (25, 30, 43, 44, 50, 57–64) and proteins (65–70) have also been previously reported as biomarkers in COVID-19 studies. Furthermore, these nine proteins showed strong positive relationships with COVID-19 severity scores across all original samples, with a mean correlation value of 0.579, and IL-15 representing the strongest predictor (*r* = 0.68, *p* = 2.3E−14, **Supplementary Table 4B**), which is consistent with the report of IL-15 being an indicator of fatal outcomes (71).

### 3.5 UAMS validation cohort

#### 3.5.1 UAMS cohort demographics

The UAMS validation cohort included 94 individuals who tested positive for SARS-CoV-2. The validation participants were classified into mild (*N* = 48, 54.2% female), moderate (*N* = 30, 36.7% female), and severe (*N* = 16, 62.5% female) (**Supplementary Table 1**). There is no information provided about health or vaccination status for the validation participants. Age was a significant factor for comparisons between severe vs. mild and severe vs. moderate groups, and sex and race were not significant between any validation cohort groups.

#### 3.5.2 Evaluation of discovery UTHSC DEMs and DEPs in the UAMS cohort

miRNA and protein expression data collected from the UAMS cohort were utilized to independently verify the DEMs, DEPs, and putative miR–protein relations originally discovered in the UTHSC cohort (**Supplementary Table 9**). When applying the same significance criteria (FC > 2.0, *p*-value <0.05, CV < 50%) to the validation dataset, 221 of the 273 original severe vs. mild DEMs remained significant, with 218 (98.64%) showing expression changes in the same direction as the discovery dataset. For severe vs. moderate comparison, 41 of the 195 original DEMs were significant, with 22 (53.66%) maintaining the same directional change. Similarly, 26 of 84 original moderate vs. mild DEMs were validated as significant, with 22 of these 26 (84.62%) showing consistent directional expression changes. Overall, 249 out of original 365 unique DEMs (in any of the three comparisons) from the discovery dataset were validated as significant in the validation dataset. Across all three comparison groups, miR-130-3p was the only DEM that achieved statistical significance in the validation set. In contrast, 15 DEMs were significantly significant across all comparison groups in the discovery UTHSC dataset (**Figure 3A**). Partial validations, let-7e-5p, let-7i-5p, and miR-1289, were significant in both the severe vs. mild and moderate vs. mild comparisons, but in the severe vs. moderate comparison, while they showed significant *p*-values (<0.05), fold changes were below the 2.0 threshold.

Detailed evaluation of the original DEPs in the validation dataset is provided in **Supplementary Table 10**. By applying the same criteria (FC > 1.5, *p*-value <0.05, CV < 50%) to the UAMS cohort, 556 of 742 (74.93%) severe vs. mild DEPs remained significant, with all 556 showing the same expression changes as observed in the UTHSC discovery dataset. For severe vs. moderate comparisons, 290 of 503 (57.65%) original DEPs were validated as significant, with 287 of these 290 (98.97%) maintaining consistent directional changes. Among moderate vs. mild comparisons, 24 of 69 (34.78%) original DEPs were significant in the validation set, with 24 of the 24 showing the same directional expression pattern. Overall, 585 out of the original 801 unique DEPs (in any of the three comparisons) from the discovery dataset were validated as significant in the validation dataset. In the validation dataset, 13 DEPs, i.e., PINLYP, FDX1, GBP2, IL-1RL1, IL-6, MAD1L1, MYBPC1, MYL3, MYLPF, NADK, SFRP1, SMOC1, and ZBTB16, were significant in all three comparison groups and demonstrated consistent directional change relative to the discovery cohort (**Figure 3B**). There were two additional DEPs with partial validation: DNPER was significant in severe vs. mild and severe vs. moderate and moderate vs. mild comparison; however, the FC threshold was not met in the latter comparison, and CHCHD10 was significant by FC and *p*-value for all three comparison groups, but its CV (50.29%) exceeded the CV acceptance criterion (50%) and therefore not classified as significant.

#### 3.5.3 Pathway enrichment comparisons with the UAMS cohort

Pathway enrichment comparisons between the original UTHSC cohort (737 DEPs), and the 553 DEPs common between the two cohorts are shown in **Supplementary Table 11** and lists the 32 pathways shown in **Figure 4** and the additional 29 pathways shown in the Sankeys (**Figure 5** and **Supplementary Figure 2**). With a few exceptions, the 10 enriched pathways in the discovery dataset were significant in the UAMS cohort and the common DEPs subset from the validation set. **Supplementary Table 11** shows the *p*-value, *z*-score, and number of significant proteins in the pathway for the original and validation cohorts. The validation cohort using the common protein or all significant has significant minus log10 *p*-value >3.0 and *z*-score >2.0 for all 32 pathways. The top 10 pathways in the original cohort are in the top 12 pathways for the common protein in the validation cohort. The pathways using the common 553 proteins had an average deviation in the −log10 *p*-value of 0.84 with four pathways (Neuroinflammation Signaling, Pulmonary Fibrosis Idiopathic Signaling, Role of Hypercytokinemia/Hyperchemokinemia in the Pathogenesis of Influenza, and Signaling by Rho Family GTPases) with a −log10 *p*-value that is less than 4. These results confirm that the biological processes/pathways identified in the discovery cohort are reproducible between severe vs. mild COVID-19 severity across independent patient populations.

#### 3.5.4 Confirmation of UTHSC miR–protein pairs in UAMS cohort

Pearson correlations were conducted to validate miR–protein connections identified in the UTHSC discovery set (shown in **Figure 5** and **Supplementary Figure 2**) using the DEMs and DEPs identified and measured in the UAMS cohort. Complete correlation comparisons between datasets are provided in **Supplementary Table 12**. The validation analysis demonstrated strong consistency between cohorts, with average miR–protein correlations of −0.339 in the validation set compared to −0.334 in the discovery dataset. Among the 122 miR–protein pairs shown in **Figure 5**, when analyzed, 81 pairs (66%) showed correlation differences of less than 0.15 between datasets, and 71 pairs (58%) exhibited stronger negative correlations in the UAMS cohort than the UTHSC discovery dataset. A correlation plot comparison of UTHSC discovery miR–protein relationships (*y*-axis) with corresponding UAMS validation correlations (*x*-axis) for all 122 miR–protein connections is found in **Figure 6** and showed a significant positive correlation (*r* = 0.43, *p* < 0.0001). Several miR–protein pairs demonstrated robust negative correlations across both cohorts. For example, miR-30d-5p with tumor necrosis factor super family 10B (TNFRSF 10B) and miR-199a-5p with hepatocyte growth factor (HGF) kept strong negative correlations (*r* < −0.5) in both datasets. Another 26 miR–protein pairs showed significant correlations below 0.40 across datasets including the following: miR-199a-5p with rho guanine nucleotide exchange factor 5 (ARHGEF5; discovery, *r* = −0.49, *p* = 7.00E−07; validation, *r* = −0.51, *p* = 1.65E−07), let-7e-5p with c-c chemokine family number 7 (CCL7; discovery *r* = −0.46, *p* = 4.03E−06; validation, *r* = −0.50, *p* = 3.73E−07), miR-30d-5p with c-jun (JUN; discovery, *r* = −0.47, *p* = 2.15E−06; validation, *r* = −0.55, *p* = 1.09E−08), and miR-29c-3p with tumor necrosis super family 1A (TNFRSF1A; discovery, *r* = −0.59, *p* = 4.98E−10; validation, *r* = −0.48, *p* = 1.02E−06). Notably, the miR-6871-5p-protokineticin 1(PROK1) showed weak, non-significant correlations in both cohorts, suggesting that this may represent a false-positive prediction by IPA.

**Figure 6:**
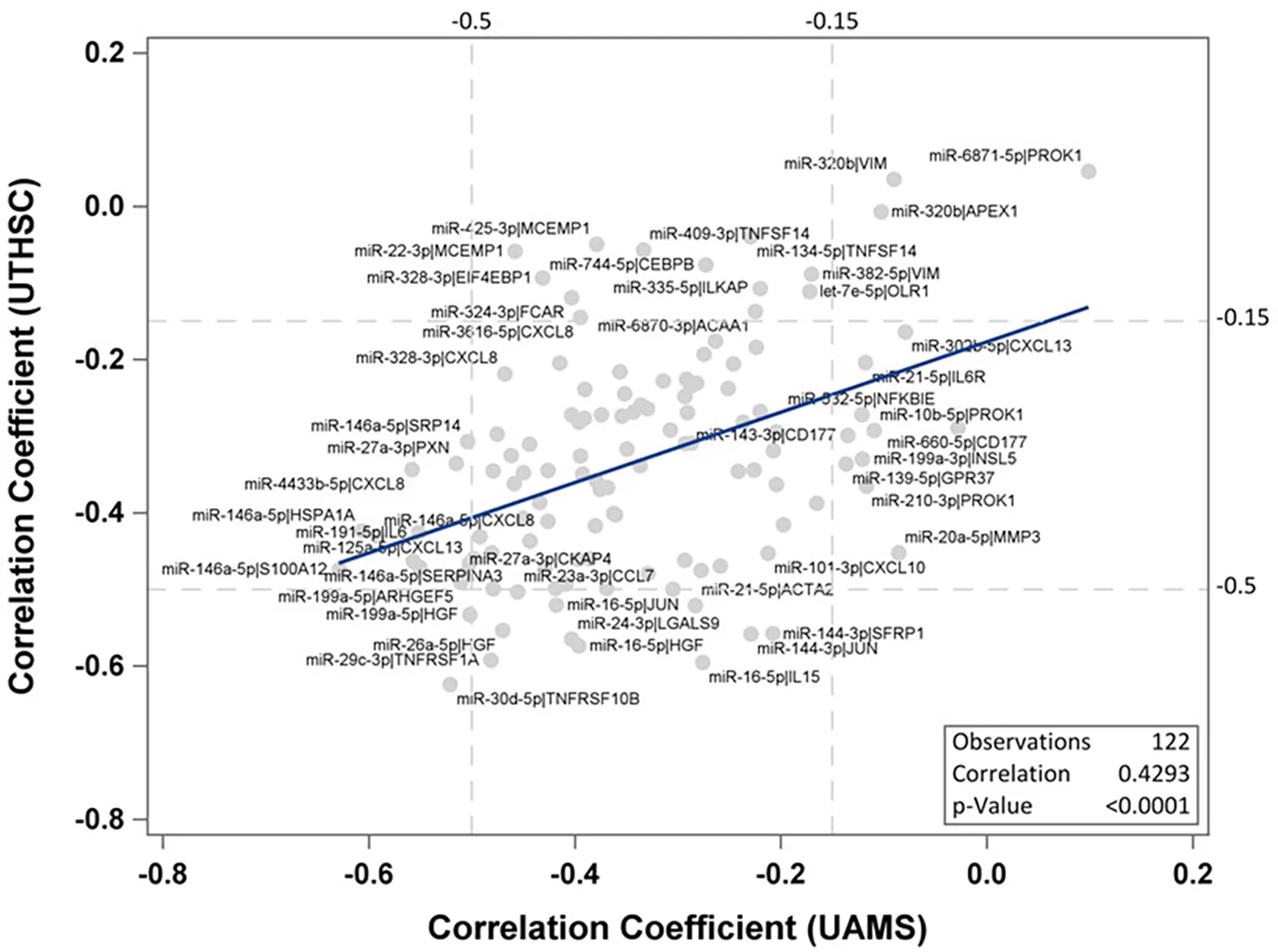
Comparison of UTHSC vs. UAMS miR–protein correlations shown in Sankey of IPA prediction (**Figure 5**). Correlation data are provided in **Supplementary Table 12**.

## 4 Discussion

This study aimed to discover circulating miR and protein biomarkers that predict COVID-19 response severity while enhancing our understanding of disease mechanisms. Previous studies examining either miRs or plasma proteins left significant knowledge gaps in our understanding of how miRs regulate disease progression. To address these limitations, we developed an integrated analytical approach that combines miR target prediction with protein expression data, as illustrated in **Figure 1**. Our findings carry important diagnostic and therapeutic implications. Several identified miRs and proteins show strong potential as biomarkers for COVID-19 response severity, and miR–protein pairs with inverse changes in severe patients could serve as prioritized candidates for future functional investigation and therapeutic hypothesis generation (72–74).

The validity of our findings is strongly supported by their broad concordance with prior COVID-19 miRNA and proteomic literature, while the integrated analytical framework reveals novel regulatory relationships that extend beyond what either data type could provide alone. The majority of our top severity-associated miRNA signals confirm previously reported biomarkers, including miR-17-5p, miR-20a-5p, miR-106a-5p, miR-451a (42), and miR-150-5p (*r* = −0.62) as a marker of severe pulmonary disease (48), and miR-342-3p and miR-191-5p showing negative associations with severity consistent with independent reports (49). At the protein level, IL-6 appearing in 9 of the 10 most significant pathways is among the most extensively reported severity and mortality predictors in COVID-19 (44–46), and additional pro-inflammatory, endothelial injury, and pulmonary remodeling markers including CXCL8, CXCL10, IL-1RN, SDC1, KRT19, MSLN, and EPPK1 confirm severity-associated signals previously characterized in independent cohorts (35, 51–54). At the pathway level, neutrophil degranulation (*z*-score >6) and pathogen-induced cytokine storm signaling have both been prominently reported as drivers of COVID-19 severity (7–9, 55, 56, 75). To facilitate interpretation of the complex miR–protein networks identified in **Figure 5** and **Supplementary Figure 2**, enriched pathways were organized into four major biological modules: Cytokine & Innate Immune Signaling pathways; Chronic Inflammation, Autoimmunity & Neuroinflammation pathways; Fibrosis, Organ Pathology & Tissue Remodeling pathways; and Core Cell Signaling & Cancer Biology pathways. This higher-order organization highlights that severity-associated miR–protein interactions are not confined to isolated inflammatory mediators but instead converge on coordinated biological programs encompassing acute innate immune activation, chronic inflammatory remodeling, tissue repair and fibrosis, and fundamental cellular signaling processes. Notably, IL-6 was present in 23 of the 29 enriched pathways and contributed to pathways within all four biological modules, emphasizing its central role as a shared hub linking acute inflammatory responses with chronic tissue remodeling and broader cellular signaling programs. The predominance of Cytokine & Innate Immune Signaling pathways, together with the extensive involvement of IL-6 across pathway modules, is consistent with current models of severe COVID-19 pathogenesis and suggests that diverse manifestations of disease severity may converge on a common inflammatory network. While the majority of individual biomarkers confirm prior literature, the principal novel contribution of this work lies in the integrated miR–protein regulatory relationships most notably the miR-144-3p/SFRP1 pro-fibrotic axis and the miR-146a-5p/S100A12 NET-formation pathway, which are consistent with (76), but not derivable from prior single-omic studies. Taken together, the high degree of concordance between our findings and the existing literature validates our integrated analytical approach, while the novel miR–protein regulatory pairs provide a mechanistic framework and prioritized candidate list for future functional investigation.

Prior studies have independently profiled miRNAs or plasma proteins in patients with COVID-19 to identify severity-associated biomarkers—including small RNA-seq identification of serum miRNA signatures in severe vs. mild disease (28), large-scale proteomic characterization of inflammatory mediators (14, 16), and reports of upregulated inflammatory proteins such as HGF and IL-6 in severe COVID-19 (44, 45). To our knowledge, no published study has simultaneously profiled both large-scale miRNA and proteomic data from the same individuals to derive regulatory miR–protein relationships. Single-omic approaches, while informative, are inherently limited in their ability to resolve how miRNA expression changes translate into altered protein levels and downstream pathway dysregulation. By profiling miRNAs and proteins from the same plasma samples and integrating these data through a dual-evidence analytical framework, this study bridges that gap and moves beyond biomarker cataloguing toward a mechanistic understanding of miR-mediated regulation in COVID-19 severity.

Because miRNA–mRNA interaction prediction models face accuracy limitations (~70%) and high false-positive rates (77, 78), we used two complementary approaches to identify miR–protein regulatory relationships: IPA-based target prediction and independent Pearson correlation analysis between miR and protein expression, with false discovery minimized by applying a threshold of *r* < −0.50 (*p*-value < 3.8E−07, FDR < 2.15E−05). These methods were then applied to an independent UAMS cohort that differed demographically and clinically from the discovery dataset. The miR–protein relationships identified in the validation cohort overlapped significantly with the discovery set (**Figure 6**; *r* = 0.43, *p* < 0.0001), with 91 of 101 (90.1%) miR–protein pairs with significant negative correlations in the UTHSC cohort also significant in the UAMS cohort. Seven of the 122 miR–protein pairs showed non-significant correlations in both cohorts, potentially representing false-positive predictions or pairs where the miRNA or protein does not effectively translate into plasma, while pairs with weaker or discordant correlations across cohorts may reflect false-negative predictions or broader miR network responses.

By integrating target prediction with correlation analysis in the UTHSC dataset, this investigation identified novel miR–protein regulatory mechanisms driving COVID-19 pathogenesis and disease severity, which confirm and extend findings from previous independent studies. An example is miR-144-3p, which showed decreased plasma expression in patients with COVID-19 compared to healthy controls (29, 62, 79). Madè et al. (62) demonstrated that circulating miR-144-3p can distinguish between critical and non-critical illness in hospitalized patients, findings that align with our identification of miR-144-3p as a candidate biomarker for disease severity within our study population. Additionally, we identified one of its regulatory targets: soluble secreted frizzled-related protein 1 (SFRP1) by IPA prediction and a strong negative correlation with miR-144-3p (*r* = −0.56, *p* = 7.7E−9) (80). SFRP1 functions as an antagonist of the Wnt/β-catenin signaling pathway, which exhibits dysregulation in several chronic pulmonary diseases, including emphysema and asthma (81–83). Recent animal studies have demonstrated that elevated SFRP1 levels promote the polarization of alveolar macrophages toward an alternative Th2-inflammatory phenotype, thereby enhancing eosinophil recruitment to pulmonary tissues (84). While eosinophilia is generally considered a favorable prognostic indicator in patients with COVID-19, particularly those administered inhaled corticosteroids (85), local recruitment of eosinophils into the COVID-19 lung may paradoxically exacerbate injury through release of pro-fibrotic cytokines, potentiating fibrosis—a mechanism also observed in emphysema (82, 83). These findings suggest that the miR-144-3p/SFRP1 regulatory axis may represent a key molecular mechanism underlying COVID-19 severity, where decreased miR-144-3p expression leads to elevated SFRP1 levels and subsequent pro-fibrotic responses, further supported by the strong correlation between MSLN, an established fibrosis marker, and COVID-19 severity scores in our cohort.

In both the discovery and validation datasets, the most significant IPA pathway associated with COVID-19 severity was neutrophil degranulation. High neutrophil counts are associated with increased disease severity and poor prognosis in COVID-19 (55, 56, 61, 86, 87), and overactive production of neutrophil extracellular traps (NETs) contributes to vascular injury and thrombosis (88). miR-146a-5p, a well-established negative feedback inhibitor of pro-inflammatory NF-κB signaling through targeting of TRAF6 and IRAK1 (89), was negatively correlated with S100A12 in both study populations (discovery, *r* = −0.47, *p* = 1.86E−06; validation, *r* = −0.63, *p* = 1.46E−11). Downregulation of miR-146a-5p has been associated with increased NET formation (90), and S100A12 release from neutrophils has been shown to promote NET formation in myocardial injury (91). Although a direct regulatory relationship between S100A12 and miR-146b-5p has been established (92), miR-146a-5p and miR-146b-5p share an identical seed sequence with largely overlapping predicted targets (93, 94), suggesting that miR-146a-5p downregulation may similarly increase S100A12 release and promote excessive NET formation, contributing to the thrombotic complications of severe COVID-19 (95). Consistent with its broader role in NF-κB regulation, miR-146a-5p also demonstrated significant negative correlations with CXCL8 (discovery, *r* = −0.43, *p* = 1.85E−05; validation, *r* = −0.59, *p* = 4.23E−10) and SERPINA3 (discovery, *r* = −0.46, *p* = 3.32E−06; validation, *r* = −0.56, *p* = 7.8E−09), both regulators of neutrophil recruitment and activity (96, 97), likely through direct 3’ UTR binding or indirect suppression of NF-κB signaling. Taken together, these findings suggest that targeting miR-146a-5p regulatory networks may offer a promising avenue for reducing thrombotic risk in critically ill patients with COVID-19.

This comprehensive analysis yielded valuable insights despite several limitations in study design and sample collection. The two cohorts differ in age, race, and region, with the original cohort younger than the validation cohort across all severity groups (mild: 39.3 vs. 49.0 years; moderate: 44.6 vs. 52.3 years; severe: 52.2 vs. 72.1 years), and approximately 31% of UAMS participants were scored using an alternative severity classification schema rather than the WHO criteria applied to the full UTHSC cohort, which may have introduced phenotypic heterogeneity. This heterogeneity likely reduced quantitative agreement between cohorts and may partially explain the moderate correlation coefficient observed in **Figure 6**. Nevertheless, the high concordance in the direction and significance of many miR–protein relationships across two geographically and clinically distinct populations supports the biological reproducibility of the identified networks despite differences in classification methods. Plasma studies are inherently restricted and do not provide information on cell- and tissue-specific coverage, including immune activation, epithelial injury, endothelial dysfunction, and cell death. In addition, circulating miRNAs and proteins represent integrated systemic signals derived from multiple tissues and cell types and therefore cannot resolve cell-intrinsic regulatory programs occurring within specific immune compartments such as germinal center B cells, Tfh cells, or PBMC subsets. Consequently, the miR–protein relationships identified here should be interpreted as candidate systemic regulatory networks rather than direct evidence of compartment-specific mechanisms. Future single-cell and tissue-resolved studies will be required to define the cellular origins and functional consequences of these interactions. Participant samples were collected at enrollment on the day of diagnosis, creating temporal variability, and COVID-19 duration was estimated from self-reported symptom onset and initial positive PCR results due to unavailability of electronic medical records for many mild cases. Small sample sizes per response group (≤30) constrained the ability to assess additional clinical factors, and unlike many prior COVID-19 studies, mild infected participants rather than healthy controls served as the baseline comparator, a deliberate choice reflecting the unreliability of ICU admission and hospitalization as sole severity markers during periods of constrained healthcare capacity (35). Finally, the associations identified here are correlational and hypothesis-generating; functional validation through *in vitro* miRNA overexpression or inhibition studies and cell-resolved approaches will be necessary to establish causality and define the biological mechanisms underlying these candidate regulatory networks.

## 5 Conclusion

In this study, we conducted the first integrated analysis of circulating miRNA and protein expression from the same SARS-CoV-2-infected individuals, replicated in an independent validation cohort, to identify biomarkers and elucidate regulatory mechanisms associated with COVID-19 severity. The vast majority of identified miRNA and protein biomarkers are concordant with prior literature, lending strong face validity to our integrated analytical approach, while the novel miR–protein regulatory pairs, including the miR-144-3p/SFRP1 pro-fibrotic axis and the miR-146a-5p/S100A12 NET-formation pathway, provide mechanistic hypotheses not derivable from single-omic studies alone. The predominance of inverse miRNA–protein correlations observed in plasma supports the biological plausibility of miRNA-mediated regulation and demonstrates that integrated multiomic profiling can move beyond biomarker cataloguing to generate testable mechanistic insights. These findings provide a molecular framework for understanding COVID-19 pathogenesis and severity that may inform the development of diagnostic and therapeutic strategies such as SARS-CoV-2 becoming integrated into the landscape of seasonal infectious diseases. Future longitudinal studies tracking these miRNA–protein pairs over the course of infection would yield valuable insights into disease progression dynamics and further refine the candidate targets identified here.

## Data Availability

The original contributions presented in the study are publicly available. This data can be found here: https://open.fda.gov/apis/research/covidmirnaandproteomics/.
