# Supplementary material for: Integrated miR-omics and proteomics reveal the regulatory role of miR in protein networks associated with COVID-19 disease progression
Source: Front Immunol. 2026 Sep 14;17:1796638. doi: 10.3389/fimmu.2026.1796638 (PMC13616804; doi:10.3389/fimmu.2026.1796638)
Supplement: Supplementary file 1 [file SupplementaryFile1.docx]

Integrated miRNA-omics and Proteomics Reveal the Regulatory Role of miRNA in Protein Networks Associated with COVID-19 Disease Progression

Supplementary Material

1.0 Supplementary Figures

**Supplemental Figure 1**. Pearson correlations between significantly downregulated miRNAs and upregulated proteins with severity scores. Negative log10 p-value scores are plotted for (**A**) significant negatively correlated miRNAs and (**B**) significant positively correlated proteins on the y-axis according to their strength of correlation, strong; (|r|) greater than 0.5, moderate; 0.3 < (|r|) < 0.5, and weak; (|r|) < 0.3, on the x-axis. Expression levels of downregulated miRNAs and upregulated proteins identified in the severe vs mild group comparison were selected for these analyses. Significance FDR<0.05.

**Supplemental Figure 2.** A Sankey diagram illustrating significantly different (p<0.05, FC>2 in sev vs. mild comparison) miRNAs and protein with a correlation |r| > 0.5, filtered for canonical pathways which are significantly enriched with a -log10(p-value) > 4 and a z-score > 3. MiRNA-protein connections in green lines represent experimentally observed, orange lines represent high-predicted, yellow lines represent moderate-predicted by IPA, blue lines represent that no information on miRNAs-proteins association are available from IPA and UAMS |r| > 0.4 and grey lines represent that no information on miRNAs-proteins association are available from IPA and UAMS |r| < 0.4. The pathways are color coded into 4 groups: red font for Cytokine & Innate Immune Signaling pathways; blue font for Chronic Inflammation, Autoimmunity & Neuroinflammation Pathways, green font for Fibrosis, Organ Pathology & Tissue Remodeling pathways; and black font for Core Cell Signaling & Cancer Biology pathways. There are 64 miRNAs, 55 proteins and 29 pathways represented in this diagram.

**2.0 Supplementary Tables**

**Supplemental Table 1:** UAMS Validation Demographics Table

***Columns*:** Characteristic; Patient Response Category, (Mild (N=48), Moderate (N=30), Severe (N=16); Severity Comparison	(^1^P-value (severe vs mild), ^2^P-value (severe vs moderate), ^3^P-value (moderate vs mild))

**Supplementary Table 2:** MiRNA univariate statistics for each group and group comparison.

Comparison groups, Severe vs Mild, Severe vs. Moderate, Moderate vs Mild

Significant differential expression miRNAs are FC > 2.0, P value < 0.05, CV< 50%.

***Columns:*** miRNA name; N Pooled; CV Pooled; Mild Mean Norm CPM; Mild Mean log2 norm cpm; Mild SD log2 norm cpm; Moderate Mean Norm CPM; Moderate Mean log2 norm cpm; Moderate SD log2 norm cpm; Severe Mean Norm CPM; Severe Mean log2 norm cpm; Severe SD log2 norm cpm; fold ratio linear Sev vs Mild; Abs FC Sev vs Mild; raw p Sev vs Mild; fdr p Sev vs Mild; Sig FC>2 P<0.05 CV<50 Sev vs Mild; fold ratio linear Sev vs Mod; Abs FC Sev vs Mod; raw p Sev vs Mod; fdr p Sev vs Mod; Sig FC>2 P<0.05 CV<50 Sev vs Mod; fold ratio linear Mod vs Mild; Abs FC Mod vs Mild; raw p Mod vs Mild; fdr p Mod vs Mild; Sig FC>2 P<0.05 CV<50 ModvsMild

**Supplementary Table 3**: Protein univariate statistics for each group and group comparison

Comparison groups, Severe vs Mild, Severe vs. Moderate, Moderate vs Mild

Significant DEPs are FC > 1.5, P value < 0.05, CV< 50%

Protein univariate statistics for each group and group comparison

Comparison groups, Severe vs Mild, Severe vs. Moderate, Moderate vs Mild

Significant DEPs are FC > 1.5, P value < 0.05, CV< 50%

.

***Columns:*** OlinkID; UniProt: ProteinCCL; Description; Entrezgene summary; N Pooled Samples;	CV Pooled Samples; Mild Mean NPX; Mild Mean NPX log2; Mild SD NPX log2; Moderate Mean NPX; Moderate Mean NPX log2; Moderate SD NPX log2;	Severe Mean NPX; Severe Mean NPX log2; Severe SD NPX log2;	fold ratio linear Sev vs Mild; Abs FC Sev vs Mild; raw p Sev vs Mild; fdr p Sev vs Mild; Sig FC>1.5 P<0.05 CV<50 Sev vs Mild; fold ratio linear Sev vs Mod; Abs FC Sev vs Mod; raw p Sev vs Mod; fdr p Sev vs Mod;	Sig FC>1.5 P<0.05 CV<50 Sev vs Mod; fold ratio linear Mod vs Mild; Abs FC Mod vs Mild;	raw p Mod vs Mild’; fdr p Mod vs Mild; Sig FC>1.5 P<0.05 CV<50 Mod vs Mild

**Supplementary Table 4A**: List of miRNAs negatively associated with severity scores by Pearson correlation, cutoff FDR < 0.05. DEMs identified in the severe vs mild comparison group with a negative FC were used in this analysis.

***Columns:*** MiRNA; CorrCoeff Sev Score: CorrPval Sev Score: CorrFDR Sev Score: miRNAs identified in Figure 3A

**Supplementary Table 4B**: List of proteins positively associated with severity scores by Pearson correlation, cutoff FDR < 0.05. DEPs identified in the severe vs mild comparsion group with a negative FC were used in this analysis.

***Columns:*** protein; CorrCoeff Sev Score: CorrPval Sev Score; CorrFDR Sev Score; Proteins in Figure 3B

**Supplementary Table 5:** P values and z scores for IPA canonical pathways common and unique to response group comparison, severe vs mild, moderate vs. mild and severe vs. moderate comparisons.

***Columns:*** Ingenuity Canonical Pathways: moderate vs mild; -log(p-value); Ratio; z-score; Molecules; Molecule Num; Ingenuity Canonical Pathways: severe vs moderate; -log(p-value; Ratio; z-score; Molecules; Molecule Num; ;	Ingenuity Canonical Pathways: severe vs mild; -log(p-value); Ratio; z-score; Molecules; Molecule Num

**Supplementary Table 6:** MiRNA-protein target predictions from IPA target filter tool.

***Columns:*** MiRNA; Source; miRNA Confidence; ID; Protein IPA; DEM Sig FC>2 P<0.05 CV<50; DEP Sig FC>1.5 P<0.05 CV<50; Fig 5 MiRNA_COVID_References; Fig 5 Protein_COVID_References; Fig5_Sankey_MiR_Prot_122Pairs

**Supplementary Table 7:** Pearson correlations between miRNA and DEPs.

***Columns:*** MiRNA; Protein Olink; Protein IPA; Correlation Coefficient; Corr P Value; fdr p; miRNA Confidence; Canonical Pathways Top10 Per Protein

**Supplementary Table 8:** Proteins in in silico prediction and experimental correlation Sankeys from Neutrophil degranulation, Wound Healing and Cytokine Storm pathways with their list of miR pairs

***Columns:*** Canonical Pathway; Protein; MiRNA to Protein combinations from Prediction Sankey; MiRNA to Protein combinations from Correlation Sankey

**Supplemental Table 9:** UAMS validation miR data of UTHSC DEMs

***Coulmns:*** miRNA name, Group Comparison, AbsFC uthsc, raw p uthsc, CV, Pooled uthsc, AbsFC uams, raw p uams, CV Pooled uams, Sig FC2 P005 CVLT50 uams, same direction both

**Supplemental Table 10:** UAMS validation protein data of UTHSC DEPs

***Coulmns:*** OlinkID, Protein, Group Comparison, AbsFC uthsc, raw p uthsc, CV Pooled uthsc,, AbsFC uams	raw, p uams, CV Pooled uams, Sig FC15 P005 CVLT50 uams, same direction both

**Supplemental Table 11*:*** Comparison of IPA canonical pathways using UTHSC DEPs and UAMS protein data for severe vs mild

***Columns***: Canonical Pathways; UTHSC 737Prot Minuslog10pval; UTHSC 737Prot Zscore; UTHSC 737Prot Mol Num; Common UAMS UTHSC 553 Prot Minuslog10pval; Common UAMS UTHSC 553 Prot ZScore; Common UAMS UTHSC 553 Prot Mol Num

**Supplemental Table 12:** Comparison of UTHSC miR-protein correlation to UAMS miR-protein correlations in Sankey Figures

***Columns:*** protein, miRNA, mirprot, Corr Coeff UTHSC, Corr Pval UTHSC, Corr FDR UTHSC, Corr Coeff UAMS, Corr Pval UAMS, Corr FDR UAMS, In Sankey IPA (Figure5), In Suppl2 Corr, miRNA Confidence UTHSC
